# The impact of diabetes mellitus on biomarkers for the diagnosis of periprosthetic joint infection

**DOI:** 10.3389/fcimb.2025.1720431

**Published:** 2025-12-17

**Authors:** Tianmiao Cheng, Xi Chen, Hairong Ding, Cifu Qu, Jing Chen, Dong Zheng, Jun Qiu

**Affiliations:** Center of Clinical Laboratory, The First Affiliated Hospital of Soochow University, Suzhou, China

**Keywords:** diabetes mellitus, periprosthetic joint infection, aseptic loosening, C-reactive protein, CRP-albumin ratio

## Abstract

**Objective:**

The aim of this study is to explore the impact of diabetes on the diagnosis of periprosthetic joint infection (PJI) by evaluating the levels of biomarkers and their diagnostic efficacy.

**Methods:**

According to the diagnostic criteria for diabetes mellitus (DM), 394 patients were divided into the DM group (n=113) and the Non-DM group (n=281); each of these two groups was further subdivided into the PJI group and the aseptic loosening (AL) group based on the presence or absence of PJI. The levels of C-reactive protein (CRP), erythrocyte sedimentation rate (ESR), fibrinogen (FIB), D-dimer, CRP-albumin ratio (CAR), and CRP-FIB ratio (CFR) were collected from all patients. Receiver operating characteristic (ROC) curves were used to evaluate the diagnostic efficacy of the aforementioned biomarkers under different glucose metabolism statuses.

**Results:**

In both the DM group and the Non-DM group, the levels of all biomarkers in patients with PJI were significantly higher than those in patients with AL (P<0.001). The CAR showed the highest diagnostic efficacy in both groups (AUC = 0.918 in the DM group and AUC = 0.931 in the Non-DM group). Notably, compared with the Non-DM group, the optimal cut-off values of CRP and ESR were elevated in the DM group (CRP: 9.960 mg/L vs 7.895 mg/L; ESR: 43.0 mm/h vs 36.5 mm/h).

**Conclusion:**

Diabetes does not affect the biomarker levels in patients with PJI. However, for diabetic patients, the diagnostic thresholds of CRP and ESR are relatively higher. Thus, caution should be exercised in clinical diagnosis to avoid false positives. Among all indicators, the CAR demonstrates the best diagnostic efficacy and can serve as the preferred biomarker for PJI patients with comorbid diabetes.

## 1 Introduction

Total joint arthroplasty (TJA) is a common and effective method for treating end-stage osteoarthritis (Walker et al., 2002). With the deepening of global aging, the annual number of TJA surgeries has shown a consistent upward trend (Kapadia et al., 2016). Periprosthetic joint infection (PJI) is not only a major cause of TJA failure but also a devastating postoperative complication of TJA (Bozic et al., 2010). Although the incidence of PJI is relatively low, ranging from 0.5% to 2%, its occurrence not only causes severe damage to patients’ health but also imposes a heavy economic burden on them (Pulido et al., 2008; Akindolire et al., 2020). Previous studies have indicated that diabetes is one of the important risk factors for PJI after TJA (Wier et al., 2024). In recent years, with the annual increase in the prevalence of diabetes, the incidence of PJI is expected to rise accordingly (Zhou et al., 2024).

The prevalence of diabetes in China has shown a continuous upward trend, rising from less than 1% in 1980 to 12.4% in 2018 (Wang et al., 2021). Diabetes is not only a key risk factor for vascular and neurological diseases, but also affects the levels of various serum inflammatory biomarkers (Ismail et al., 2021; Le et al., 2025). The hyperglycemic state activates inflammatory cells, prompting them to release cytokines such as interleukin-6 (IL-6), IL-1, and TNF-α; these cytokines then stimulate the liver to synthesize and release C-reactive protein (CRP) (Stanimirovic et al., 2022). Previous studies have found that compared with the control group, the plasma fibrinogen level in diabetic patients is significantly higher (324 ± 139 mg/dl vs. 656 ± 130 mg/dl, P<0.05) (Bembde, 2012). Guo et al. discovered that the erythrocyte sedimentation rate (ESR) is independently associated with the incidence and severity of diabetic nephropathy in diabetic patients (Guo et al., 2020). In clinical practice, PJI and aseptic loosening (AL) are difficult to distinguish due to their shared clinical symptoms, such as pain and joint swelling. Therefore, accurate differentiation between PJI and AL is crucial for guiding precise treatment. In recent years, researchers have advocated for the use of techniques like polymerase chain reaction, next-generation sequencing, and mass spectrometry as novel methods for diagnosing PJI (Esteban and Gómez-Barrena, 2021; Tan et al., 2022). While these techniques can significantly improve the diagnostic accuracy of PJI, they cannot be independently performed in most hospitals and involve relatively high testing costs (Torchia et al., 2019). Currently, there is still a lack of research on the impact of diabetes on the levels and diagnostic capabilities of various biomarkers in PJI patients. Consequently, we conducted this study using traditional biomarkers (CRP and ESR), fibrinolytic biomarkers (fibrinogen [FIB] and D-dimer), and emerging ratio-based biomarkers (CRP-albumin ratio [CAR]; CRP-FIB ratio [CFR]). All these biomarkers have demonstrated favorable diagnostic value in previous studies (Tarabichi et al., 2023; Cao et al., 2025).

The primary objectives of this study include the following three aspects: (1) exploring the impact of diabetes on the levels of inflammatory biomarkers in patients with PJI; (2) identifying the optimal diagnostic biomarkers applicable to both diabetic and non-diabetic PJI patients; (3) evaluating the diagnostic efficacy of various biomarkers in the subgroups of diabetic and non-diabetic patients. We are committed to achieving these objectives to investigate the association between diabetes and serum inflammatory biomarkers in PJI patients, accurately identify effective diagnostic biomarkers for diabetic and non-diabetic patients respectively, and further conduct a detailed assessment of the diagnostic performance of these biomarkers in different subgroups.

## 2 Methods

### 2.1 Study design

This study was a single-center retrospective study, which strictly adhered to the ethical principles for medical research involving human subjects as outlined in the Declaration of Helsinki and had obtained approval from the Ethics Committee of the First Affiliated Hospital of Soochow University. For this study, we included the case data of patients who underwent revision surgery for total knee or total hip arthroplasty at our hospital from June 2019 to June 2025. To ensure the accuracy of the results, the following exclusion criteria were applied: (1) having inflammatory diseases such as rheumatoid arthritis, gout, and systemic lupus erythematosus; (2) having malignant tumors; (3) having periprosthetic fractures; (4) having prosthetic dislocations; (5) having abnormal liver function; (6) having missing data; (7) having hematological diseases; (8) recent use of anticoagulant drugs. All diabetic patients included in the study were type 2 diabetes mellitus (DM) patients. The diagnostic criteria for diabetes mellitus were as follows: (1) glycated hemoglobin level≥6.5%; (2) fasting blood glucose level≥7.0 mmol/L; (3) random blood glucose level≥11.1 mmol/L; (4) 2-hour blood glucose level in oral glucose tolerance test≥11.1 mmol/L; (5) use of hypoglycemic drugs; (6) previous history of DM. First, patients were divided into the DM group and Non-DM group according to the diagnostic criteria for DM. Then, each patient group was further divided into the PJI group and the AL group according to the diagnostic criteria. The diagnostic criteria for PJI were established according to the 2018 International Consensus Meeting (ICM) (Parvizi et al., 2018). The diagnosis of AL refers to the relevant criteria reported in previous literature (Huang et al., 2019), specifically including: (1) pain in the thigh or hip region, or knee pain; (2) radiographic evidence of prosthesis loosening, such as separation between the prosthesis components and bone tissue, displacement of the prosthesis components, or the presence of a radiolucent line; (3) negative periprosthetic culture; (4) exclusion of PJI.

### 2.2 Patient data collection

Detailed information of patients was collected via the hospital’s medical record system, including age, gender, surgical site, culture results, and preoperative levels of CRP, ESR, FIB, albumin, and D-dimer. These preoperative test indicators were measured using fasting venous blood samples collected by specialized nurses on the day of the patient’s admission or the early morning of the following day. CAR and CFR were calculated based on the above data.

### 2.3 Statistical analysis

All statistical analyses in this study were performed using IBM SPSS Statistics software (Version 21). Continuous variables were presented as mean ± standard deviation, while categorical variables were described using frequency (n) and percentage (%). For statistical tests, the Mann-Whitney U test was used for continuous variables; for categorical variables, the chi-square test or Fisher’s exact test was selected based on the data distribution. A P-value of less than 0.05 was considered statistically significant. In addition, the diagnostic value of each biomarker was evaluated using the receiver operating characteristic curve (ROC), area under the curve (AUC), and its 95% confidence interval (CI). The optimal cut-off value for each biomarker as a diagnostic tool for PJI was determined based on the Youden index. Meanwhile, the sensitivity, specificity, positive predictive value, and negative predictive value of each biomarker were calculated to comprehensively assess their diagnostic efficacy. Statistical significance: *: P<0.05, **: P<0.01, ***: P<0.001.

## 3 Results

### 3.1 Demographic characteristics and biomarker levels in DM and non-DM patients

**Figure 1:**
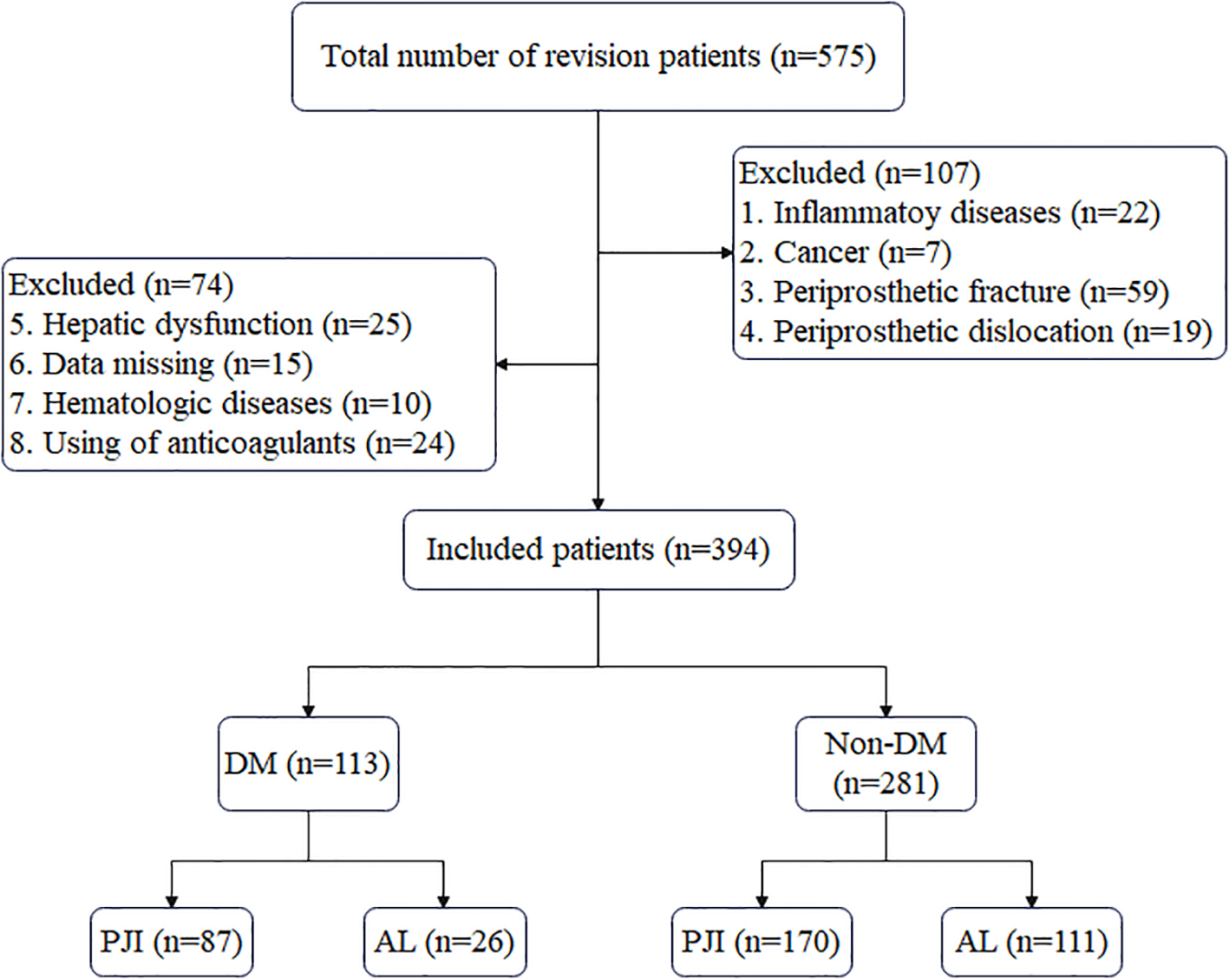
Flowchart of patient inclusion. DM, diabetes mellitus; PJI, periprosthetic joint infection; AL, aseptic loosening.

**Figure 2:**
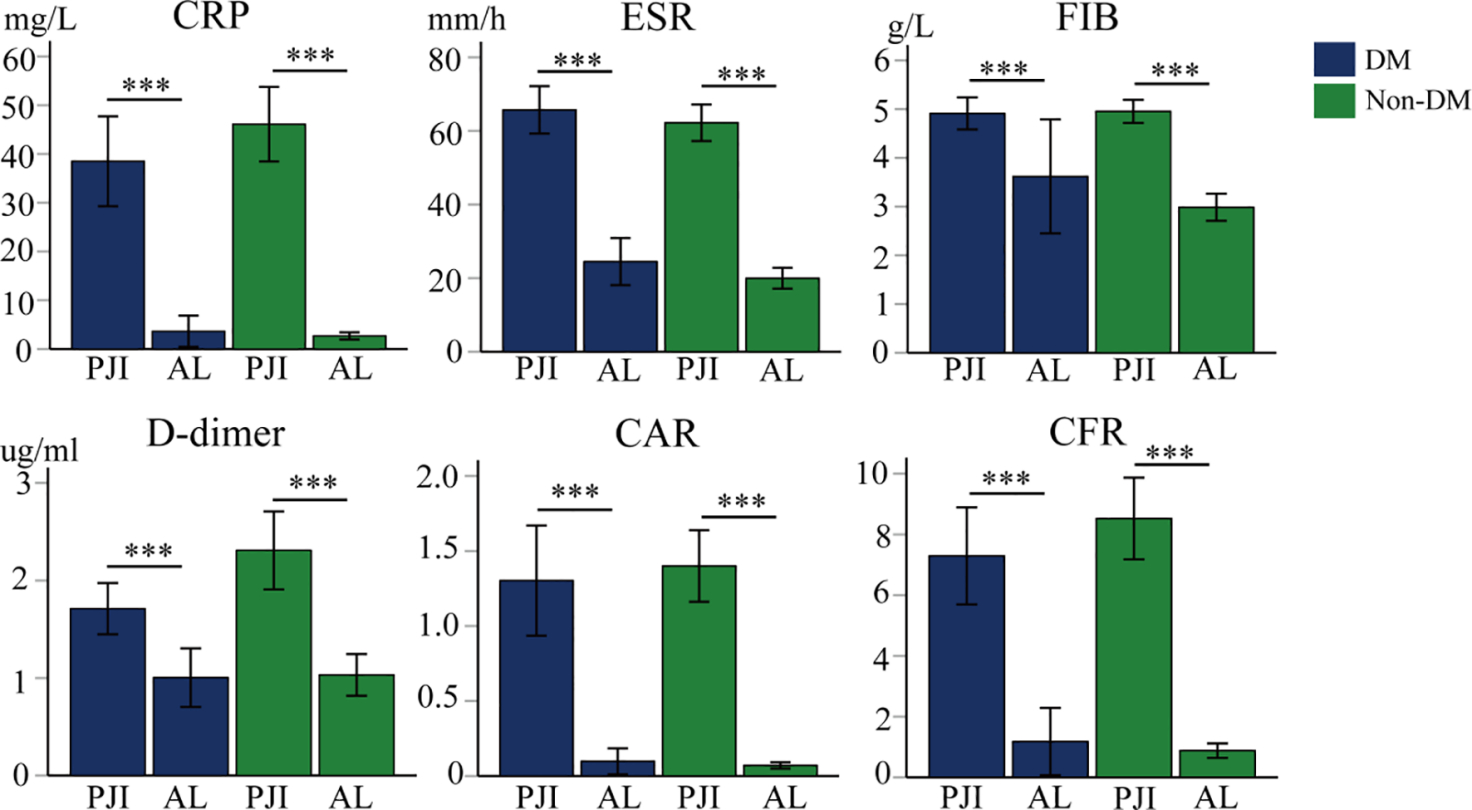
The levels of various serum inflammatory biomarkers in DM patients and Non-DM patients. CRP, C-reactive protein; ESR, erythrocyte sedimentation rate; FIB, fibrinogen; CAR, CRP-albumin ratio; CFR, CRP-FIB ratio; ***: P<0.001.

As shown in **Figure 1**, a total of 394 patients were included in the study after screening, among whom 113 were DM patients and 281 were Non-DM patients. Compared with the Non-DM group, the prevalence of PJI was higher in the DM group (60.5% vs. 77.0%), which indicates that diabetes is one of the risk factors for PJI. There were no significant differences in the age and gender distribution between PJI and AL patients in either the DM group or the Non-DM group (P>0.05). However, the incidence of knee joint infection was significantly higher than that of hip joint infection in both groups, with P = 0.035 in the DM group and P<0.001 in the Non-DM group (**Table 1**). Meanwhile, in both the DM and Non-DM groups, the levels of various biomarkers in PJI patients were significantly higher than those in AL patients (P < 0.001) (**Table 2**; **Figure 2**).

**Table 1:** Demographic characteristics.

Category	DM		P value	Non-DM		P value
	PJI (n=87)	AL (n=26)		PJI (n=170)	AL (n=111)	
Age	67.98 ± 9.64	71.38 ± 7.55	0.101	65.49 ± 11.86	64.23 ± 11.15	0.374
Gender						
male	30 (34.5)	11 (38.7)		74 (43.5)	42 (37.8)	0.344
female	57 (65.5)	15 (61.3)	0.469	96 (56.5)	69 (62.2)	
Joint						
Hip	30 (34.5)	15 (57.7)	0.035	71 (41.8)	80 (72.1)	<0.001
Knee	57 (65.5)	11 (42.3)		99 (58.2)	31 (27.9)	

DM, diabetes mellitus; PJI, periprosthetic joint infection; AL, aseptic loosening.

**Table 2:** Levels of biomarkers in DM and Non-DM patients.

Markers	DM		P value	Non-DM		P value
	PJI(n=87)	AL(n=26)		PJI(n=170)	AL(n=111)	
CRP (mg/L)	38.50 ± 43.20	3.64 ± 7.98	<0.001	46.10 ± 50.57	2.68 ± 3.87	<0.001
ESR (mm/h)	65.71 ± 30.16	24.50 ± 15.78	<0.001	62.24 ± 32.76	19.97 ± 15.15	<0.001
FIB (g/L)	4.91 ± 1.54	3.62 ± 2.89	<0.001	4.95 ± 1.57	2.99 ± 1.48	<0.001
D-dimer(ug/mL)	1.71 ± 1.23	1.00 ± 0.74	<0.001	2.31 ± 2.65	1.03 ± 1.14	<0.001
CAR	1.30 ± 1.73	0.10 ± 0.22	<0.001	1.40 ± 1.58	0.07 ± 0.11	<0.001
CFR	7.29 ± 7.51	1.18 ± 2.74	<0.001	8.52 ± 8.90	0.88 ± 1.27	<0.001

DM, diabetes mellitus; PJI, periprosthetic joint infection; AL, aseptic loosening; CRP, C-reactive protein; ESR, erythrocyte sedimentation rate; FIB, fibrinogen; CAR, CRP-albumin ratio; CFR, CRP-FIB ratio.

### 3.2 The diagnostic value of biomarkers in DM patients with PJI

**Figure 3:**
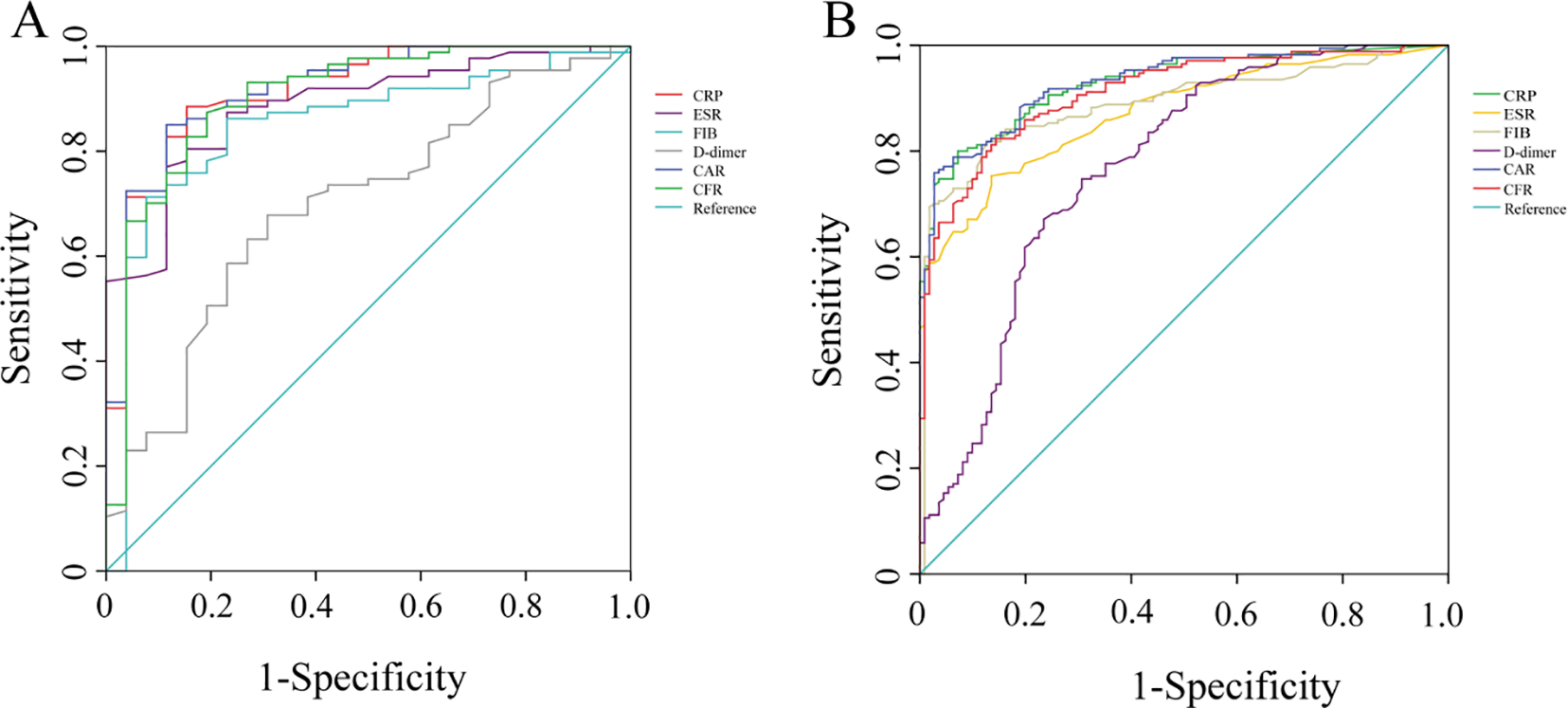
The ROC curves of CRP, ESR, fibrinogen, D-dimer, CAR, and CFR in DM and Non-DM patients. **(A)** DM group; **(B)** Non-DM group. CRP, C-reactive protein; ESR, erythrocyte sedimentation rate; FIB, fibrinogen; CAR, CRP-albumin ratio; CFR, CRP-FIB ratio.

In the DM group, ROC analysis results showed that the AUC values of CRP, ESR, FIB, D-dimer, CAR, and CFR were 0.915 (95% CI 0.852-0.978), 0.886 (95%CI 0.820-0.951), 0.849 (95%CI 0.760-0.937), 0.698 (95%CI 0.585-0.810), 0.918 (95%CI 0.857-0.980), and 0.902 (95%CI 0.827-0.977), respectively. Among these biomarkers in the DM group, CAR exhibited the highest diagnostic value for PJI, with an optimal cut-off value of 0.134, a sensitivity of 85.1%, and a specificity of 88.5%. It was followed by CRP, which could diagnose PJI with an optimal cut-off value of 9.960 mg/L, a sensitivity of 88.5%, and a specificity of 84.6%. D-dimer had the poorest diagnostic performance, with an AUC value of only 0.698 (95%CI 0.585-0.810) (**Table 3**; **Figure 3A**).

**Table 3:** The diagnostic value of biomarkers in DM patients with PJI.

Markers	AUC	95% CI	Youden index	Optimal cutoff value	Sen (%)	Spec (%)	PPV (%)	NPV (%)
CRP (mg/L)	0.915	(0.852, 0.978)	0.731	9.960	88.5	84.6	95.06	68.75
ESR (mm/h)	0.886	(0.820, 0.951)	0.655	43.0	77.0	88.5	95.71	53.49
FIB (g/L)	0.849	(0.760, 0.937)	0.635	4.05	71.3	92.3	96.88	48.98
D-dimer (ug/mL)	0.698	(0.585, 0.810)	0.370	0.94	67.8	69.2	88.06	39.13
CAR	0.918	(0.857, 0.980)	0.735	0.134	85.1	88.5	96.10	63.89
CFR	0.902	(0.827, 0.977)	0.681	1.048	87.4	80.8	93.83	65.63

DM, diabetes mellitus; PJI, periprosthetic joint infection; CRP, C-reactive protein; ESR, erythrocyte sedimentation rate; FIB, fibrinogen; CAR, CRP-albumin ratio; CFR, CRP-FIB ratio; CI, confidence interval; Sen, sensitivity; Spec, specificity; PPV, positive predictive value; NPV, negative predictive value.

### 3.3 The diagnostic value of biomarkers in non-DM patients with PJI

In the non-DM group, ROC analysis results showed that the AUC values of CRP, ESR, FIB, D-dimer, CAR, and CFR were 0.928 (95%CI 0.900-0.957), 0.873 (95%CI 0.833-0.912), 0.889 (95%CI 0.850-0.929), 0.769 (95%CI 0.710-0.828), 0.931 (95%CI 0.903-0.958), and 0.911 (95%CI 0.878-0.944), respectively. Consistent with the DM group, CAR demonstrated the highest diagnostic value for PJI in the non-diabetic group, with an optimal cut-off value of 0.232, a sensitivity of 75.9%, and a specificity of 97.3%. It was followed by CRP, which could diagnose PJI with an optimal cut-off value of 7.895 mg/L, a sensitivity of 80.0%, and a specificity of 92.8%. Again, D-dimer had the poorest diagnostic performance, with an AUC value of 0.769 (95%CI 0.710, 0.828) (**Table 4**; **Figure 3B**).

**Table 4:** The diagnostic value of biomarkers in Non-DM patients with PJI.

Markers	AUC	95% CI	Youden index	Optimal cutoff value	Sen (%)	Spec (%)	PPV (%)	NPV (%)
CRP (mg/L)	0.928	(0.900, 0.957)	0.728	7.895	80.0	92.8	94.44	75.18
ESR (mm/h)	0.873	(0.833, 0.912)	0.618	36.500	75.3	86.5	89.51	69.57
FIB (g/L)	0.889	(0.850, 0.929)	0.692	3.695	81.8	87.4	90.85	75.78
D-dimer (ug/mL)	0.769	(0.710, 0.828)	0.441	0.955	74.7	69.4	77.44	64.17
CAR	0.931	(0.903, 0.958)	0.732	0.232	75.9	97.3	97.71	72.00
CFR	0.911	(0.878, 0.944)	0.679	1.638	82.4	85.6	89.74	76.00

DM, diabetes mellitus; PJI, periprosthetic joint infection; CRP, C-reactive protein; ESR, erythrocyte sedimentation rate; FIB, fibrinogen; CAR, CRP-albumin ratio; CFR, CRP-FIB ratio; CI, confidence interval; Sen, sensitivity; Spec, specificity; PPV, positive predictive value; NPV, negative predictive value.

Synthesizing the above results, we found that compared with the Non-DM group, the optimal cut-off values of CRP and ESR were higher in the DM group (CRP: 9.960 mg/L vs. 7.895 mg/L; ESR: 43.0 mm/h vs. 36.5 mm/h). This result indicates that diabetes can affect the diagnostic threshold for PJI. Meanwhile, D-dimer exhibited the poorest diagnostic performance in both the DM and Non-DM groups, with the lowest diagnostic sensitivity and specificity.

### 3.4 Comparison of biomarker levels within the subgroup

Based on stratification by culture results and the site of the affected joint, we conducted intragroup comparisons of biomarker levels in DM and Non-DM patients with PJI, respectively. The results showed that except for the level of FIB being higher in PJI of the knee than in PJI of the hip (P = 0.047), no significant differences in biomarker levels were observed within all other stratified subgroups (regardless of whether patients had concurrent diabetes mellitus or not) (P>0.05) (**Table 5**). This result suggests that the aforementioned biomarkers still possess stable diagnostic value even when there are differences in culture results or the site of the affected joint.

**Table 5:** Comparison of biomarkers in the diabetic and non-diabetic PJI subgroup.

Markers	DM					
	Culture positive	Culture negative	P value	Hip	Knee	P value
	PJI(n=72)	PJI(n=15)		PJI(n=30)	PJI(n=57)	
CRP(mg/L)	35.22 ± 35.65	54.28 ± 68.53	0.311	35.79 ± 38.30	39.93 ± 45.83	0.673
ESR(mm/h)	67.86 ± 30.38	55.40 ± 27.77	0.147	69.97 ± 29.11	63.47 ± 30.71	0.343
FIB(g/L)	4.94 ± 1.57	4.79 ± 1.45	0.747	4.46 ± 1.41	5.15 ± 1.57	0.047
D-dimer(ug/mL)	1.66 ± 1.22	1.94 ± 1.32	0.432	1.36 ± 0.96	1.90 ± 1.32	0.055
CAR	1.15 ± 1.44	2.01 ± 2.68	0.080	1.17 ± 1.42	1.37 ± 1.88	0.617
CFR	6.82 ± 6.36	9.58 ± 11.59	0.197	8.49 ± 8.65	6.67 ± 6.83	0.285

Markers	Non-DM					
	PJI(n=135)	PJI(n=35)	P value	PJI(n=71)	PJI(n=99)	P value
CRP(mg/L)	47.38 ± 50.58	41.14 ± 50.97	0.517	45.31 ± 47.17	46.66 ± 53.10	0.864
ESR(mm/h)	63.60 ± 32.11	56.97 ± 35.11	0.287	66.20 ± 33.70	59.39 ± 31.93	0.182
FIB(g/L)	5.00 ± 1.61	4.77 ± 1.41	0.435	4.81 ± 1.61	5.06 ± 1.54	0.300
D-dimer(ug/mL)	2.21 ± 2.67	2.69 ± 2.54	0.343	2.19 ± 3.11	2.39 ± 2.27	0.627
CAR	1.45 ± 1.59	1.22 ± 1.56	0.462	1.39 ± 1.48	1.40 ± 1.65	0.968
CFR	8.76 ± 9.16	7.61 ± 7.87	0.499	9.24 ± 9.74	8.018.26	0.377

DM, diabetes mellitus; PJI, periprosthetic joint infection; CRP, C-reactive protein; ESR, erythrocyte sedimentation rate; FIB, fibrinogen; CAR, CRP-albumin ratio; CFR, CRP-FIB ratio; statistical significance: P<0.05.

## 4 Discussion

Given the increased incidence of PJI after TJA in diabetic patients, we compared the diagnostic value of traditional biomarkers (CRP and ESR), fibrinolytic biomarkers (FIB and D-dimer), and emerging ratio-based biomarkers (CAR and CFR) between diabetic and non-diabetic patients. The results showed that compared with the AL group, the levels of serum inflammatory biomarkers in diabetic patients with PJI were significantly higher. Among all biomarkers, CRP and CAR were the two optimal indicators for diagnosing PJI in both diabetic and non-diabetic patients. Notably, the optimal cut-off value of CRP was higher in the DM group than in the Non-DM group, whereas the optimal cut-off value of CAR was higher in the Non-DM group than in the DM group. This result emphasizes that the impact of diabetes must be fully considered when using CRP and CAR to diagnose PJI, thereby avoiding false positives and false negatives, respectively. Subgroup analysis results indicated that neither bacterial culture results nor the site of PJI had a significant impact on serum biomarker levels.

CRP is a traditional and highly sensitive inflammatory biomarker, which is mainly synthesized by the liver under stimulation by proinflammatory cytokines (Pepys and Hirschfield, 2003). Previous studies have shown that as the level of glycated hemoglobin (HbA1c) increases, the probability of a rise in CRP concentration increases significantly (King et al., 2003). Meanwhile, CRP has also been confirmed as a potent predictor of cardiovascular diseases (Yosef-Levi et al., 2007). ESR is a non-specific inflammatory biomarker (Ghanem et al., 2009). When inflammation occurs in the body, it stimulates the aggregation of red blood cells, which in turn leads to an increase in ESR. Results from previous studies indicated that for every 1% increase in HbA1c level in diabetic patients, ESR increases by 2.3 mm/h (Fiorentino et al., 2015). These findings confirm the close association between blood glucose control and systemic inflammatory response in diabetic patients, and further highlight the importance of establishing individualized diagnostic thresholds for CRP and ESR in diabetic patients. The results of our study showed that the serum levels of CRP and ESR in AL patients with diabetes were higher than those in AL patients without diabetes. At the same time, ROC analysis results revealed that the optimal cut-off values of CRP and ESR for diagnosing PJI were higher in the DM group than in the Non-DM group. Therefore, when diagnosing PJI in the diabetic population, it is necessary to appropriately increase the diagnostic thresholds of CRP and ESR to effectively reduce the false-positive rate, thereby achieving a balance between diagnostic specificity and sensitivity. We also need to note that even if the CRP level is lower than the aforementioned proposed cut-off value, it does not necessarily completely rule out infection. Given that patients with diabetes are a high-risk group for PJI, increasing the diagnostic threshold may contradict the clinical practice principle of “lowering the diagnostic threshold for high-risk populations”. Therefore, in clinical practice, it is necessary to comprehensively determine the diagnosis by integrating clinical manifestations, imaging signs, and microbiological evidence in accordance with diagnostic criteria.

As an acute-phase protein, FIB is not only a key factor in the blood coagulation process but also closely associated with the body’s infectious status (Chandy et al., 2017). Previous studies have confirmed that FIB exhibits favorable diagnostic efficacy for PJI (Song et al., 2023). Although D-dimer is commonly used for screening venous thromboembolism (VTE), multiple studies have suggested its potential value in PJI diagnosis, making it a promising diagnostic biomarker (Bounameaux et al., 1994; Shahi et al., 2017). Additionally, other studies have verified that diabetes can cause vascular endothelial damage, which in turn activates the coagulation pathway and leaves patients in a pathological hypercoagulable state (Rao et al., 1999). The results of our study showed that the FIB level in AF patients with diabetes was higher than that in AL patients without diabetes; however, there was no difference in the FIB level between diabetic patients with PJI and non-diabetic patients with PJI. This phenomenon may be attributed to the fact that the baseline FIB level in diabetic patients is already elevated. As a result, when PJI occurs, the dynamic changes in FIB levels are masked by the high baseline value, ultimately leading to its diagnostic value for PJI being weaker than that of CRP and CAR.

Albumin is a negative acute-phase protein synthesized by the liver. Under inflammatory conditions, the liver prioritizes the synthesis of positive acute-phase proteins such as CRP and FIB, which in turn leads to a reduction in albumin synthesis (Castell et al., 1990). Additional studies have shown that in diabetic patients, low plasma albumin levels are associated with elevated HbA1c levels (Bhonsle et al., 2012). Therefore, for diabetic patients, the CAR, in the form of a ratio, not only integrates inflammation- and nutrition-related information but also reduces the interference of albumin levels on inflammation assessment results. The results of our study showed that CAR exhibited the highest diagnostic value for PJI in both the DM and Non-DM groups, which is consistent with the conclusions of previous studies (Wu et al., 2023; Shi et al., 2025). Our study also found that, similar to non-diabetic PJI patients, diabetic PJI patients had a higher proportion of knee joint infections, and this result is consistent with the findings of prior research (Henry et al., 2019). It is worth noting that we found the cut-off value of CAR for diagnosing PJI in patients with diabetes was lower than that in non-diabetic PJI patients (0.134<0.232), and this trend is completely opposite to that of CRP. We speculate the mechanism as follows: On one hand, Diabetes can reduce the baseline level of albumin (Sasatomi et al., 2012); on the other hand, with the same increase range of CRP, the lower albumin (as the denominator) will amplify the inflammatory signal, enabling CAR to reach the threshold for significantly distinguishing PJI from AL earlier. Therefore, the diagnostic cut-off value of CAR for diabetic PJI patients decreases. This result also indicates that CAR can more comprehensively adapt to the metabolic and inflammatory characteristics of diabetic patients.

Our study classifies patients with PJI based on whether they have concurrent diabetes mellitus, and evaluates the diagnostic efficacy of traditional biomarkers, fibrinolytic biomarkers, and other inflammatory biomarkers. However, this study still has limitations. First, it is a single-center retrospective study, which inevitably introduces inherent data bias. Second, the sample size of our study is limited, especially for patients with AL in the DM group. Therefore, subsequent studies should include a larger sample size and adopt a prospective study design to further verify the reliability and generalizability of the conclusions of this study, and provide more sufficient evidence support for clinical practice.

## 5 Conclusion

In summary, this study found that diabetes can affect the levels of diagnostic biomarkers for PJI and their diagnostic cut-off values. Further analysis confirmed that CRP and the CAR are the optimal biomarkers for diagnosing PJI in diabetic patients, and the diagnostic value of both is not affected by the site of joint involvement or the results of pathogenic bacteria culture. The above findings provide a reliable evidence-based basis for the accurate diagnosis of PJI in patients with diabetes, and more in-depth studies are still needed in the future to further improve the PJI diagnostic system for this population.

## Data Availability

The original contributions presented in the study are included in the article/supplementary material. Further inquiries can be directed to the corresponding authors.
